# Supplementary material for: Comparative multiomics analysis of cell physiological state after culture in a basket bioreactor
Source: Sci Rep. 2022 Nov 23;12:20161. doi: 10.1038/s41598-022-24687-4 (PMC9686226; doi:10.1038/s41598-022-24687-4)
Supplement: Supplementary file 1 — Supplementary Information 1. [file 41598_2022_24687_MOESM1_ESM.zip › raw data/Transcriptomic raw data/1.QC/1.Error/TE1_1_Error.pdf]

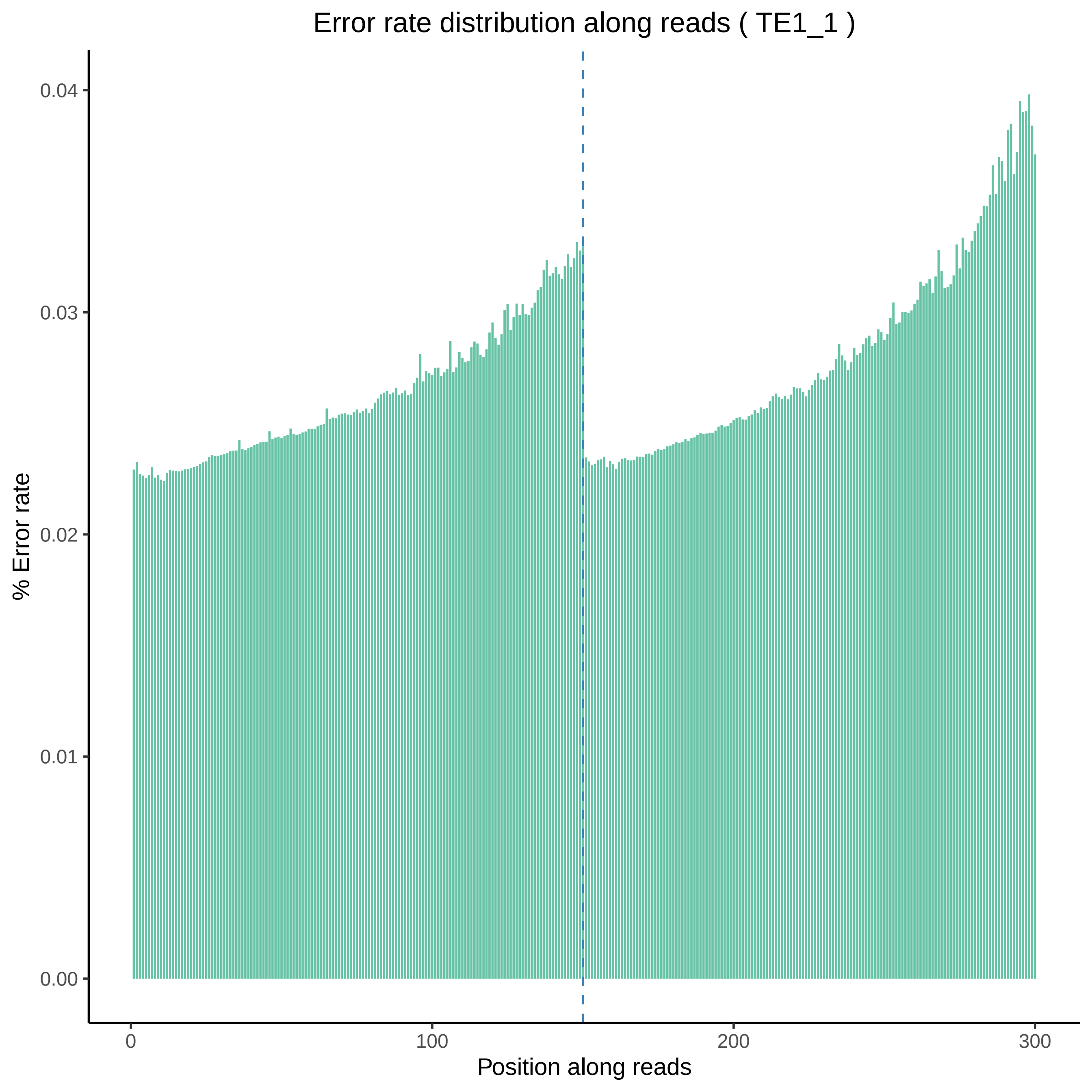
Error rate distribution along reads (TE1_1)
% Error rate
0.00
0.01
0.02
0.03
0.04
0
100
200
300
Position along reads
